# Hepatocyte Lysosomal Membrane Stabilization by Olive Leaves against Chemically Induced Hepatocellular Neoplasia in Rats

**DOI:** 10.4061/2011/736581

**Published:** 2010-12-05

**Authors:** N. M. Abdel-Hamid, M. A. El-Moselhy, A. El-Baz

**Affiliations:** ^1^Department of Biochemistry, College of Pharmacy, Minia University, Minia, Egypt; ^2^Department of Pharmacology, College of Pharmacy, Minia University, Minia, Egypt; ^3^Department of Medical Biochemistry, College of Medicine, Mansura University, Mansura, Egypt

## Abstract

Extensive efforts are exerted looking for safe and effective chemotherapy for hepatocellular carcinoma (HCC). Specific and sensitive early biomarkers for HCC still in query. Present work to study proteolytic activity and lysosomal membrane integrity by hepatocarcinogen, trichloroacetic acid (TCA), in Wistar rats against aqueous olive leaf extract (AOLE).TCA showed neoplastic changes as oval- or irregular-shaped hepatocytes and transformed, vesiculated, and binucleated liver cells. The nuclei were pleomorphic and hyperchromatic. These changes were considerably reduced by AOLE. The results added, probably for the first time, that TCA-induced HCC through disruption of hepatocellular proteolytic enzymes as upregulation of papain, free cathepsin-D and nonsignificant destabilization of lysosomal membrane integrity, a prerequisite for cancer invasion and metastasis. AOLE introduced a promising therapeutic value in liver cancer, mostly through elevating lysosomal membrane integrity. The study substantiated four main points: (1) the usefulness of proteolysis and lysosomalmembrane integrity in early prediction of HCC. (2) TCA carcinogenesis is possibly mediated by lysosomal membrane destabilization, through cathepsin-D disruption, which could be reversed by AOLE administration. (3) A new strategy for management of HCC, using dietary olive leaf system may be a helpful phytotherapeutic trend. (4) A prospective study on serum proteolytic enzyme activity may introduce novel diagnostic tools.

## 1. Introduction

The metabolism of functional proteins is a continuous process involving proteolysis, which is the destruction of individual protein molecules and their replacement through protein synthesis. Intracellular proteolysis includes either lysosomal (cathepsins) or nonlysosomal pathway, which is mostly executed by calpains and proteasomes [1–3]. The rate of degradation of both intracellular and extracellular proteins is greatly dependent on cathepsin activity. 

The degradation rate of proteins can be monitored by measuring cathepsin activity oscillations [4]. Lysosomes are membrane-bound structures containing hydrolytic enzymes capable of degrading most of the cellular constituents. They also play a pivotal role in secretion and transport processes. Leakage of lysosomal enzymes accounts for many tissue derangements and target organopathies [5, 6]. Lysosomes are found in all animal cells and are more numerous in disease-fighting cells.

Lysosomal enzyme disorders contribute to several human diseases, either due to genetic defects in its enzyme expression or the escape of lysosomal enzymes (lysozymes) into extralysosomal medium [7]. Hepatocellular carcinoma (HCC) is considered as one of the worst prognostic cancers in the world. It develops mostly over chronic liver diseases of cirrhotic fate. HCC nodules are always encapsulated by excessive extracellular matrix (ECM) materials among a bed of cirrhotic tissue [8, 9]. ECM degradation by specific proteases was reported to play an important role in cancer invasion and metastasis [10]. It was noticed that some herbals could ameliorate anticancer-induced lysosomal abnormalities, conserving lysosomal integrity, probably, through an antioxidant mechanism [6].

Studies of the trichloroethylene (TCE) metabolites, dichloroacetic acid (DCA), trichloroacetic acid (TCA), and chloral hydrate suggested that both DCA and TCA are involved in TCE-induced liver tumorigenesis and that many DCA effects are consistent with conditions that increase the risk of liver cancer [11–13]. 

Olive tree (Olea europaea, Oleaceae) is a longevous plant, anciently known in the Mediterranean basin [14]. Olive leaves are rich in active constituents showing medicinal value. They possess many water-soluble compounds of antioxidant and anti-inflammatory properties [15]. The most important bioactive compounds are oleuropein (13.4%) and rutin (0.18%), luteolin-7-glucoside, verbascoside, apigenin-7-glucoside, hydroxytyrosol, and some other hydrolytic products, both of glycosidic and flavonoid nature [16, 17]. The leaves also contain many terpenoids having hepatoprotective and cancer-preventive potential [18]. Besides, Guinda et al. [19] had described many liposoluble principles in olive leaf, as squalene, carotenoid, vitamin E, alcohols, and saturated hydrocarbons. Many other ingredients were studied in olive leaf of different medicinal and nutritional values [20]. It is now established that olive polyphenols exert chemopreventative effects in the large intestine by interacting with signaling pathways responsible for colorectal cancer development [21]. The present work was executed to explore the possible therapeutic value of an aqueous extract of olive leaves which possess polyphenolic flavonoids, taking into account the cost-effectiveness of olive leaves as a nonordinarily utilized part and to try its efficacy in hepatocarcinoma treatment, through monitoring the early variations in liver tissue proteolytic activity, as specific enzyme, pepsin, trypsin, papain, and cathepsin enzyme activities. The total/free cathepsin activity ratio was considered as an index to lysosomal membrane integrity.

## 2. Materials and Methods

### 2.1. Materials

#### 2.1.1. Animals

Male Wistar rats weighing 190 ± 10 g were used in this experiment. These rats were given ordinary rodent diet and water ad libitum. They were housed in polyethylene cages in a humid room with controlled 12 hours light and 12 hours dark cycle. They were classified into 4 groups (8 rats/group).

#### 2.1.2. Chemicals

All chemicals were supported from local suppliers, all were of analytical grade. Tyrosine was purchased from Merck.

#### 2.1.3. Aqueous Olive Leaves Extract (AOLE)

It was kindly given by Dr. Ashraf El-Bassiouny, Natural Product Chemistry Department, Institute of Education and Science, Beni Suef University, Egypt. It was prepared by drying for 8 hours, at 40°C, water-washed fresh olive leaves collected on February of 2007, milled, homogenized and then extracted by heating with distilled water with stirring at 40°C for 48 hours as 10 g powder of dried leaves/60 CC solvent, cooled, filtered, and the supernatant was kept at 4°C during the period of administration [22]. This extract is dark-green in color, clear, fragrant, and has a little sweet taste.

#### 2.1.4. Drug Administration Schedules per Groups

The first group of rats served as control, in which animals were given 0.5 ml physiological saline by intraperitoneal route (IP), daily, for one month. The second group served as liver cancer group, given a daily IP dose of trichloroacetic acid (TCA) (Sigma-Aldrich, USA), for 5 consecutive days, by gavage as 500 mg/kg body weight, previously dissolved in distilled water, neutralized by NaOH to a pH of 5–7.5 [23]. Animals of the third group were given AOLE (softened by drying under vacuum) by gavage in a single daily dose for one month as 0.5 g/kg body weight [24]. This group was considered as positive drug control. 

Animals of group four were given doses of TCA for 5 consecutive days, 24 hours after the last dose, AOLE doses were given as stated before. Twenty-four hours after each dosing period, animals were killed, livers were excised, washed with saline, frozen directly into liquid nitrogen and kept frozen at −80°C right at the day of analytical investigations.

### 2.2. Methods

(1) Extraction of the target enzymes from liver tissue: 300 mg of liver tissue was homogenized with 6 ml ice-cold distilled water as described elsewhere [25]. Total protein was determined in each enzyme substrate prior to enzyme assessment, by the method Lowry et al. [26].

(2) Determination of specific proteolytic enzyme activity was achieved in the tissue extract on a substrate containing 1% egg albumin as the substrate protein for enzyme actions [27]. Colorimetric estimation of tyrosine, as the product of proteolysis was conducted by diluted Folin-Ciocalteu (FC) reagent, [28] against tyrosine standard curve [29].

(3) Estimation of pepsin, trypsin, and papain proteolytic activity was accomplished through changing the pH of the protein substrate per each target enzyme as stated by Anson [27], then, tyrosine was estimated as mentioned earlier [25, 29].

(4) Estimation of lysosomal membrane integrity: this variable relied on determination of cathepsin proteolytic activity in the tissue extract by acidifying the protein substrate (free cathepsin-D activity) and after dilution of the extract, then, frequent cycles of freezing and thawing were applied (total cathepsin-D activity) [30–33]. Enzyme activity in all cases was considered as *μ*g-released tyrosine/mg substrate protein. Total/free cathepsin D activity was considered as a measure of lysosomal membrane integrity [31, 32].

(5) Statistical analysis: the presented data were expressed as mean ± SE. Statistical significance was examined by one-way analysis of variance (ANOVA), [34]. *P* values less than  .05 were assumed to be statistically significant.

(6) Liver samples were stained by Hematoxylin and Eosin, examined microscopically to follow tissue changes in response to TCA and AOLE therapy.

## 3. Results

**Figure 1:**
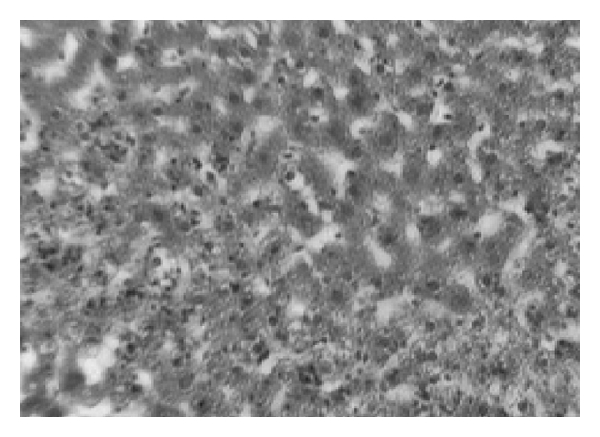
Liver section in control animals showing normal parenchymal cells with granulated cytoplasm and normal architecture.

**Figure 2:**
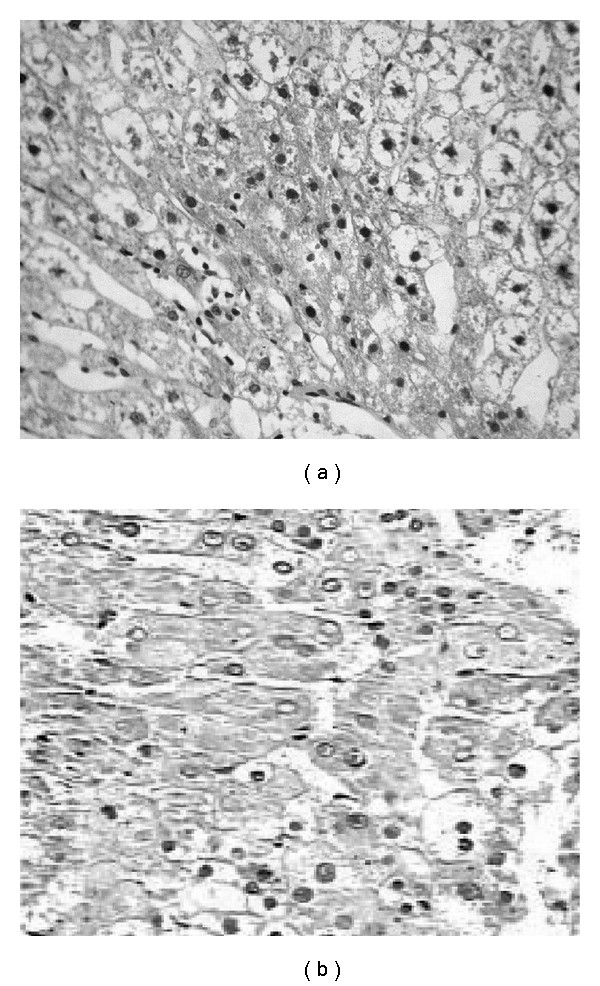
TCA-treated rats showing loss of normal architecture with oval- or irregular-shaped hepatocytes, denser nuclear chromatin, the ratio of nucleus to cytoplasm was increased, many transformed liver cells were frequently binucleated and hyperchromatic (basophilic).

**Figure 3:**
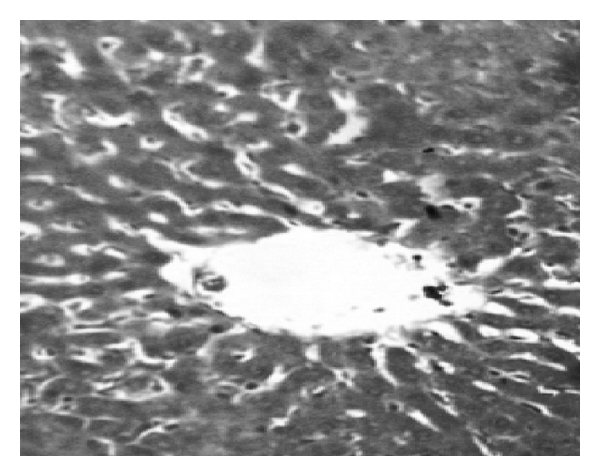
Liver section in AOLE-treated rats shows a normal central vein and blood sinusoids.

**Figure 4:**
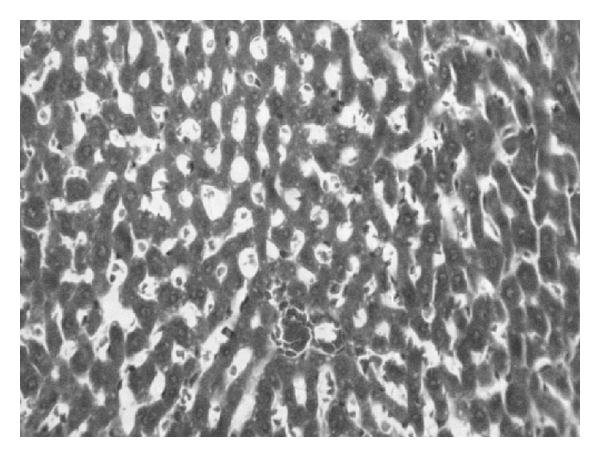
Liver section in TCA- and AOLE-treated rats showing a moderate improvement in hepatocellular structure, evidenced as a moderate improvement in vacuolation and compactness of hepatocytes compared to TCA-treated group but dilated sinusoids are still present.

TCA administration showed loss of normal architecture with oval- or irregular-shaped hepatocytes. Many transformed liver cells of foci were substantially enlarged, largely vesiculated and frequently binucleated, which were clearly distinguishable from the surrounding normal parenchyma. The nuclei were mostly found to be pleomorphic and hyperchromatic. These changes were reduced by the treatment with AOLE (Figures 1, 2, 3, and 4).

The biochemical results of the experiment are summarized in Table 1. As shown, TCA treatment significantly upregulated both papain and free cathepsin-D and non-significantly elevated specific enzyme, pepsin, trypsin, and total cathepsin-D activities, with nonsignificant reduction of lysosomal membrane integrity.

Administration of AOLE alone did not significantly affect the studied variables, but only significantly upregulated papain activity. Treatment of TCA-injured rats with AOLE significantly elevated trypsin and papain, with reduction of pepsin activities. It significantly decreased free cathepsin-D activity, leading to significant increase in lysosomal integrity index.

## 4. Discussion

Lysosomal proteolytic activity in liver cells plays a decisive role in protein metabolism, with cathepsin-A and cathepsin-D being the main enzymes involved in catabolic processes [35]. Tumor cells require specific proteolytic enzymes for invasion and metastasis, including lysosomal peptidases- cathepsin [36]. 

Recently, it was noticed that proteases play a critical role not only in tumor cell invasion, but also in the earliest stages of carcinogenesis and its associated changes: angiogenesis and metastasis. Proteases are also signaling molecules that modulate other molecules by underlying pathways in addition to their degradative role [37].

 In the present work, TCA treatment depicted nonsignificant upregulations in specific enzyme, pepsin, trypsin, and total cathepsin-D activities, with decrease in lysosomal integrity; however, it significantly activated both papain and free cathepsin-D activity. Specific proteolytic enzymes, including endopeptidases are necessary prognostic indicators (prognosticators) after tumor resection. They always rise up in patients at risk of recurrence [38]. TCA is one of three metabolites of trichloroethylene, a common solvent known classically to pollute groundwater. They are nongenotoxic carcinogens [23]. TCA is also considered as a member of peroxisome proliferators, responsible for many mutations, leading to diverse-target organ cancers [3].

This early upregulatory impact of TCA on proteolytic enzymes may contribute to the mechanism of cancer induction and invasion by this substance, an action that was noticed in some previous publications [39, 40]. Administration of AOLE alone maintained some proteolytic enzyme activities within normal values, except an elevation of papain activity to a significant level. The modulatory action on both total and free cathepsin-D activities could preserve lysosomal membrane integrity, thus protecting cellular biomolecules. This lysosomal-stabilizing potential of AOLE is mostly referred to its potent antioxidant and anti-inflammatory properties [16].

 It is clear in these results that disruption of lysosomes will lead to increased proteolytic activity, while preservation will keep cellular components and protect against neoplastic invasiveness [41]. In addition, AOLE, when given after TCA challenge, corrected the proteolytic action and restabilized lysosomal membrane to a significant value. It seems that the main therapeutic pathway for this leaf extract is mediated by pepsin and cathepsin-D variation in favour of lysosomal membrane stabilization. It is well established that cancer can induce enzyme synthesis to an extent of leakage into pericellular components [42]. However, addition of AOLE may induce increased proteolytic enzymes that might hydrolyse angiogenic proteins useful for de novo cancer progression [37].

The use of olive leaves introduces supplementary treatment in cancer management, instead of cytotoxicity and cytodestruction induced by chemotherapeutic agents. It may modulate the cellular phenotypic characteristics at end-stage of adult cells leading to programmed cell death (apoptosis) [43]. Recent study proved the usefulness of olive leave extract in inhibition of differentiation of human leukemia cell line [44].

It can be concluded that olive leaf consumption has health benefits in favouring liver cancer management. It can be recommended as a dietary supplement during liver cancer management and even after cancer protocol is stopped to guarantee recurrence. TCA can be considered as a carcinogen of proteolytic potential, by which it initiates hepatocarcinoma. Early changes in lysosomal membrane integrity, manifested by increased release of proteases may add a new line of research to figure out more specific and sensitive early biomarkers as diagnostic tools. This change in proteolytic activity necessitates further studies on blood to assess a possible escape of these studied enzymes into circulation. This may contribute to a new trend, both in early HCC assessment and prognosis.

## Figures and Tables

**Table 1:** Proteolytic enzyme activities (*μ*g-released tyrosine/mg substrate protein) and lysosomal integrity (total/free cathepsin-D activity) in liver tissue of rats treated by aqueous olive leaf extract (for one month) after trichloroacetic acid carcinogenic challenge (for 5 days). Values are expressed as mean ± S.E (*N* = 8).

	Group			
Parameter	Control	TCA (500 mg/kg)	AOLE (500 mg/kg)	TCA + AOLE (500 mg/kg + 500 mg/kg)
Specific enzyme activity	24.2 ± 0.55	24.9 ± 0.32	24.4 ± 0.39	24.7 ± 0.3
Pepsin activity	15.5 ± 0.13	15.8 ± 0.13	15.3 ± 0.13	12.3** ± 0.25
Trypsin activity	11.3 ± 0.3	11.9 ± 0.2	11.4 ± 0.6	13.3** ± 0.2
Papain activity	18 ± 1.1	22* ± 1.3	25** ± 0.9	24** ± 0.3
Free cathepsin-D activity	5.1 ± 0.1	6.2** ± 0.2	5.5 ± 0.2	4.5* ± 0.1
Total cathepsin-D activity	3.6 ± 0.13	4 ± 0.05	4 ± 0.05	4.4** ± 0.04
Lysosomal integrity	0.72 ± 0.03	0.65 ± 0.02	0.74 ± 0.03	0.99** ± 0.04

*Significantly different from control at **P < .05. Significantly different from control at *P* < .01.
